# Prenatal Exposure to Imidacloprid Affects Cognition and Anxiety-Related Behaviors in Male and Female CD-1 Mice

**DOI:** 10.3390/toxics13110918

**Published:** 2025-10-27

**Authors:** Colin Lee, Jessica Quito, Truman Poteat, Vasiliki E. Mourikes, Jodi A. Flaws, Megan M. Mahoney

**Affiliations:** 1 Comparative Biosciences, College of Veterinary Medicine, University of Illinois Urbana Champaign, Urbana, IL 61801, USAtpoteat@wisc.edu (T.P.); mourike2@illinois.edu (V.E.M.); jflaws@illinois.edu (J.A.F.); 2 Neuroscience Program, University of Illinois Urbana Champaign, Urbana, IL 61801, USA

**Keywords:** neonicotinoid, imidacloprid, cognition, anxiety, nicotinic acetylcholine receptors, developmental, sex differences

## Abstract

Neonicotinoid pesticides, including imidacloprid (IMI), are widely used in agriculture and as household insecticides. IMI displays strong affinity for insect nicotinic acetylcholine receptors (nAChRs); however, neonicotinoids still partially bind to mammalian nAChRs. Relatively little is known about how neonicotinoid exposure alters learning, memory or mood, even though nAChRs play a role in these mechanisms. We tested the hypothesis that developmental exposure to IMI impairs performance on memory tasks, and anxiety- and depressive-like behavior. We orally dosed pregnant CD-1 mice from gestation day 10 to birth with vehicle or IMI at 0.5 mg/kg/day or 5.7 mg/kg/day. When exposed animals were adults, we examined cognitive and emotional behaviors and we examined the effect of IMI on α7 and α4 nAChR subunit mRNA expression using qPCR. For both sexes, IMI exposure was associated with impaired striatal-dependent procedural learning task and hippocampal-dependent spatial learning but had no effect on hippocampal-dependent working memory. Males, but not females, displayed increased anxiety-like behavior, with low dose subjects displaying more pronounced effects, suggesting a non-linear dose response. In males, we found lower α7 subunit mRNA expression in the hippocampus and amygdala and lower α4 mRNA expression in the striatum compared to controls. Thus, exposure to IMI during a critical period is associated with disruptions to cognitive and anxiety-like behaviors. Additionally, in males, IMI exposure is associated with reduced expression of nAChR subunits in relevant brain regions.

## 1. Introduction

Neonicotinoids are pesticides that are derivatives of nicotine, and act as ligands for nicotinic acetylcholine receptors [1]. Multiple neonicotinoids are used throughout the world including acetamiprid, clothianidin, thiamethoxam, dinotefuran, and imidacloprid (IMI). These display strong affinity for insect nicotinic acetylcholine receptors (nAChRs) [2], and through this binding can cause either fatal neuroexcitation or paralysis. Meanwhile, neonicotinoids display poor affinity for vertebrate nAChRs [3], and thus have been considered relatively safe pesticides. Neonicotinoids are used heavily, with application on an estimated 79–100% of domestic corn and 34–44% of domestic soybeans in 2011 [4], and they are used in most topical flea and tick medicines [5]. Unsurprisingly, this leads to environmental contamination, as neonicotinoids are widely found in soil, dust, produce, and groundwater [6,7,8,9]. Strikingly, neonicotinoid concentrations in urban streams can be as high or higher than streams near agricultural areas [10,11], underscoring the contribution of topical pesticides and residues on food to environmental contamination [12]. Additionally, conventional wastewater treatment methods do little to remove neonicotinoids [13,14], so they are prevalent in finished drinking water [7,9]. In humans, these pesticides are found in child, adolescent, and adult samples including urine, breastmilk and blood [15,16,17,18,19].

Despite their presumed safety for mammalian exposure, a growing body of evidence demonstrates toxic effects of neonicotinoid pesticides [20,21]. Specifically, in mice and rats, IMI causes oxidative stress [22,23], central nervous system and liver damage [24,25], male and female reproductive toxicity [26,27,28], hormone changes [26,27,29,30,31], and impaired immune function [25,32]. In mice, developmental exposure to IMI is associated with changes in neurogenesis and microglia activity in the brain [23,33]. Additionally, IMI has greater effects on mammalian receptors, including human receptors, than initially thought [34]. IMI can still bind to and activate mammalian nAChRs [35], and IMI metabolites can be far more potent than their parent compounds, with agonist abilities comparable to that of nicotine [34]. nAChRs strongly regulate learning, memory, anxiety, and attention, and chronic nAChR activation by nicotine significantly alters these behaviors and nAChR function [36,37], so similar activation by neonicotinoids may cause profound cognitive and behavioral dysfunction.

Consistent with its widespread environmental contamination, IMI is found in samples from pregnant women [38,39,40], indicating prenatal exposure in humans. This period is critical for normal brain development, and perturbations during it can cause lasting behavioral consequences. Prenatal exposure to numerous drugs and environmental toxicants [41,42], including nicotine, adversely affects adult cognition. Even without adult exposure, prenatal nicotine exposure causes reductions in memory and acetylcholine levels [43], hippocampal glutamatergic signaling [44], α7 nAChR subunit expression [45], and striatal dopamine release [46]. Studies have investigated behavioral effects of prenatal IMI exposure, revealing several sensorimotor deficits [23,47], procedural memory task deficits [48] and a reduction in contextual memory in a fear conditioning task [41]. Almost no work has examined the effects of prenatal IMI exposure on mood. However, mice exposed to other neonicotinoids can have an increase in anxiety-like behavior [49,50,51,52], although this result is not always found [53]. Further, a relatively small amount of work on neonicotinoids has examined sex differences [52,54] despite the fact that in humans, urinary concentrations of neonicotinoids differ in men compared to women making this an area needing investigation [55,56]. Our work will extend these studies by examining how developmental exposure to IMI impacts anxiety-like behavior and sex differences in responsiveness.

This study examined the effects of prenatal exposure to IMI, the most commonly used neonicotinoid, on adult cognitive and mood behaviors. We tested the hypothesis that prenatal IMI exposure would be associated with impaired hippocampal- and striatal-dependent learning in a sex specific manner via altered nAChR mRNA expression. Our work focuses solely on the gestational period in contrast to similar studies [41]. We demonstrate impairments in spatial and procedural learning and increases in anxiety-like behaviors. Additionally, we find that IMI affects expression of α7 and α4 nAChR subunit expression, adding to our understanding about the potential mechanisms underlying behavioral impairment.

## 2. Materials and Methods

### 2.1. Animals and Dosing

Adult CD-1 mice were acquired from Charles River Laboratories (Boston, MA, USA). Animals were group housed at 25 °C in conventional polystyrene cages on a 12 h light/12 h dark cycle. Single males were paired with two females each, and females were checked daily for sperm plugs. Once sperm plugs were detected females were housed singly. Oral dosing began 10 days after plug formation (corresponding to gestational day 10) and continued daily until birth. Dams were randomly assigned to be given one of three doses: vehicle (corn oil with 2% DMSO, Sigma Aldritch, St. Louis, MO, USA), low dose (0.5 mg IMI/kg BW/day in vehicle, IMI from Sigma Aldrich), and high dose (5.7 mg IMI/kg BW/day in vehicle). The lower dose of IMI is within human exposure limits, and has been shown to have reproductive toxicity [30], and the higher dose is the NOAEL (no observable adverse effects limit) for mammals [17,57].

We did not measure IMI in the brain or serum of dosed dams or exposed mice as its disposition following dosing is known. In rats and mice, oral dosing with this pesticide leads to detectable IMI in the brain and urine [58,59]. IMI is detectable in mouse embryos 1 h following dosing of the pregnant mouse dam [59], and in the brains of pups nursing from dams exposed daily to IMI [33]. However, IMI is not found in the bodies of pups exposed to IMI on gestation day 9 and collected at birth [59], or in the brains of 70-day-old animals that were exposed during gestation and nursing [33]. Thus, IMI crosses the placenta but does not accumulate in tissues [59]. Based on these studies, we assume IMI was not present in the brains of our adult animals prenatally exposed to IMI.

Dams were dosed daily by gently pipetting the solution into the cheek area of the animal. Pups were weaned 21 days after birth. Only 1 male and 1 female were used from each litter for a given task. Sample sizes in behavioral tasks ranged from 4 to 12 per group and previous research shows this is appropriate for detecting differences in behavior. Sample sizes in the qPCR experiment ranged from 3 to 8. Littermates were used in different tasks. Different mice were used on each task so that mice were naïve to testing. When prenatally exposed animals were adults (>60 days of age) we tested them on a battery of behavioral tasks: the Barnes maze, rewarded alternation on the T-maze, Elevated Plus maze, Light-Dark transition test, tail suspension test, and Y-maze. All tests were done in the light phase of the light/dark cycle at 25 °C. We randomized the order of animals being tested in each experiment; researchers were not blinded during testing. All but rewarded alternation on the T-maze were analyzed with Ethovision XT-16 software (Noldus Information Technology, Wageningen, The Netherlands). All experiments with animals were approved by the Institutional Animal Care and Use Committee at the University of Illinois and adhered to ARRIVE’s guidelines for reporting animal research.

### 2.2. Barnes Maze (Spatial Learning)

The Barnes maze apparatus consisted of a circular platform (90 cm diameter) with 20 evenly spaced holes, elevated 90 cm off the ground. The maze was enclosed by a black folding screen (50 cm away) with 3 large, monochromatic shapes spaced evenly along the screen, and floodlights spaced evenly above the screens (illumination of approximately 3000 lux on the maze). For each mouse, one hole was randomly selected as the goal hole, and an escape hatch was attached underneath the hole. Mice were given apparatus habituation on day 1 and trained for days 1–4 (day 1 included both habituation and training), followed by a probe trial that occurred on day 5. For habituation, mice were placed in the escape hatch for 1 min, then moved to the maze center, and given up to 5 min to locate and enter the escape hatch; if mice did not enter the hatch in this time, they were guided to it. Training consisted of 4 trials per day. For training, mice were placed in the maze center and given up to 3 min to enter the escape hole. If they failed to enter it, they were gently guided to it and moved into the hatch. Loud white noise (90 db) was played while mice were on the maze and turned off as soon as they entered the hatch-whether by themselves or after guiding and mice remained in the hatch for 15 s. For the probe trial, the hatch was removed, and mice were allowed to explore the maze for 60 s. First hole poked, time to locate the site of the hatch and distance traveled before locating the site of the hatch were recorded. For analysis, tracking was terminated once mice located the escape hatch, as mice would occasionally locate the escape hatch but choose not to enter it, a phenomenon reported by [60].

### 2.3. T-Maze (Procedural Learning)

The maze structure had a start arm (39 cm L × 10 cm W × 20 cm H) that included a 9 cm entry zone and 2 choice arms (30 cm L × 10 cm W). Guillotine doors allowed the entry zone and choice arms to be closed off, and a 17 cm long central partition separated the choice arms. Testing was done in an illuminated room (250–300 lux). Mice were trained according to published protocols [61,62] with 1 day of habituation and 3 days of training prior to a testing day. The day prior to habituation, mice were food restricted overnight. The following day, mice were allowed to run freely through the maze for 3 min (repeated 4 times), with continuous access to food reward (50% sweetened condensed milk [SCM]) pipetted at the end of both goal arms. Each training day consisted of 11 trials, beginning with a single forced trial, with only 1 (randomly selected) goal arm open, containing 40 μL SCM pipetted at the end of the arm. In trials 2–11, both arms were open, and mice were rewarded with SCM if they selected the opposite arm as before. If they selected the incorrect arm, they were confined to the arm for the duration normally required to consume the SCM. After each trial mice were picked up and returned to the start arm. On day 5 mice were tested: there was no forced trial as in training, but otherwise the protocol was the same. Performance for a training day or testing day was quantified as the percentage of correct choices for the final 10 trials, and then normalized to the first day of training.

### 2.4. Elevated Plus Maze (Anxiety-like Behavior)

The elevated plus maze (EPM) used a plus-shaped structure elevated 50 cm off the ground, with 15 cm high walls on 2 arms and no walls on 2 arms. The arms were 35 cm-long. Mice were placed in the maze center facing an open arm and allowed to move freely for 5 min. This was done in an illuminated room (250–300). Total number of entries and time spent in open and closed arms were calculated. Increased preference for closed arms indicated greater anxiety-like behavior.

### 2.5. Light-Dark Transition Test (Anxiety-like Behavior)

The light–dark transition test used a box split between a brightly illuminated (250 lux) open chamber (28 × 28 × 31 cm) and an enclosed dark (<10 lux) chamber (14 × 14 × 31 cm) with a doorway in between. Mice were placed in the dark half and allowed to move freely through the box for 10 min. The time spent in the open half and latency to enter the open half were recorded. Increased time spent in the dark portion of the box was considered greater anxiety-like behavior.

### 2.6. Tail Suspension Test (Depression-like Behavior)

This test measured depressive-like behavior in mice [63]. Animals were habituated to the testing room for 1 h. Then, they were removed from their home cage and they were suspended in the air by taping the end of their tail to the edge of a shelf. This was done in a lit room (250–300 lux). Animals were recorded for 6 min and we scored the amount of time animals were immobile as a marker of depressive-like behavior.

### 2.7. Y-Maze (Working Memory)

The Y-maze consisted of 3 equal-sized arms (20 cm L × 7 cm W × 15 cm H) arranged in a Y shape. Mice were placed in the center of the maze and allowed to run freely through it for 10 min. We scored the last 8 min of the test. Entries into each arm were recorded, and the frequency of spontaneous alternations, defined as successive entry into each of the 3 arms without doubling back, was quantified. This test was run in a lit room (250–300 lux).

### 2.8. mRNA Expression of α4 and α7 Receptor Subunits of nAChRs in the Brain

We used quantitative PCR (qPCR) to analyze the mRNA expression levels of α4 and α7 receptor subunits of nAChRs. RNA transcripts from the hippocampus, striatum, and amygdala were analyzed because these brain areas regulate spatial memory, procedural memory, and emotion and anxiety, respectively. The animals that were run on the Barnes maze were used for these experiments. Mice (7–8 months old) were euthanized with isoflurane and decapitated. Brains were removed and flash frozen on dry ice, then stored at −80 °C (males: n = 3 control, 5 low dose, 7 high dose; females: n = 4 control, 6 low dose, 8 high dose). For tissue collection, brains were allowed to partially thaw, cut into 1 mm sections, and then bilateral punches were collected from the hippocampus (0.5 mm), striatum (1.0 mm), and amygdala (0.25 mm). Immediately following collection, RNA was extracted using a Qiagen RNeasy Mini Kit (Qiagen, Germantown, MD, USA), according to manufacturer instructions. RNA was eluted in RNAse-free water, stored at −20° C. Next, the concentration of RNA was quantified using a Thermo Scientific NanoDrop One Microvolume UV-Vis Spectrophotometer (Thermo Fisher Scientific, Waltham, MA, USA), and 200 ng RNA was reverse transcribed to cDNA with iScript Reverse Transcriptase (Bio-Rad Laboratories, Hercules, CA, USA) via a Biometra Thermocycler (Avantor, Radnor, PA, USA). Finally, qPCR was performed in 10 μL reaction volumes, containing cDNA derived from 10 ng total RNA, 0.75 pmol/μL of each forward and reverse primers, and 6.25 μL SsoFast EvaGreen dye (Bio-Rad Laboratories). Forward and reverse primers for α4 receptor subunit were ATGTCACCTCCATCCGCATC and TGACTGCAAAGTCCCCGTC, respectively (Integrated DNA Technologies, Coralville, IA, USA). Forward and reverse primers for α7 receptor subunit were CCGTGTACTTCTCCCTGAGC and TTCACTCCGGGGTACTCAGA, respectively [64]. The housekeeping gene used to normalize expression was β-actin and the forward and reverse primers were GGGCACAGTGTGGGTGAC and CTGGCACCACACCTTCTAC, respectively. Quantification was performed using the CFX96 Real-Time Detection System (Bio-Rad Laboratories, Hercules, CA, USA) and CFX Manager 3.1 Software. The qPCR protocol began with incubation at 95 °C for 5 min. This was followed by 40 cycles at 95 °C for 10 s, at 60 °C for 10 s, and at 72 °C for 10 s. Melting from 65 °C to 95 °C was followed by extension at 72 °C for 2 min. Melting temperature graphs, standard curves, and threshold cycle (Ct) values were acquired for each gene. We calculated fold change in the low- and high-exposed groups relative to the control group using the Pfaffl method which accounts for differences in primer efficiencies [65].

### 2.9. Statistics

Statistical analysis was performed with GraphPad Prism 10.6.1. Data were assessed for normality before analysis. Further, outliers were identified using ROUT analysis and removed (Q = 1%). Data were considered significant when *p* < 0.05. Two-way repeated measures ANOVAs with day of training and dose as the independent variables were followed with Dunnett’s multiple comparisons test to assess performance on the Barnes maze. Probe trial data were not normally distributed so they were analyzed with the non-parametric Kruskal–Wallis test. We used mixed effects models with Tukey’s post-test on the T-maze data. One-way ANOVAs with Tukey’s post-tests were used for the EPM, light–dark transition test, Y-maze, and Tail Suspension Test using dose as the independent variable. The qPCR data was analyzed with the Pfaffle method to generate relative fold change values of the treatment groups compared to the controls while accounting for differences in primer efficiencies [65]. These values were analyzed using an ANOVA when data were normally distributed or a Kruskal–Wallis test when data were not. We also analyzed the data with Cohen’s D analyses to determine effect sizes [66,67]. Males and females were analyzed in separate analyses.

## 3. Results

### 3.1. IMI Exposure Impairs Spatial Learning in Both Males and Females

**Figure 1:**
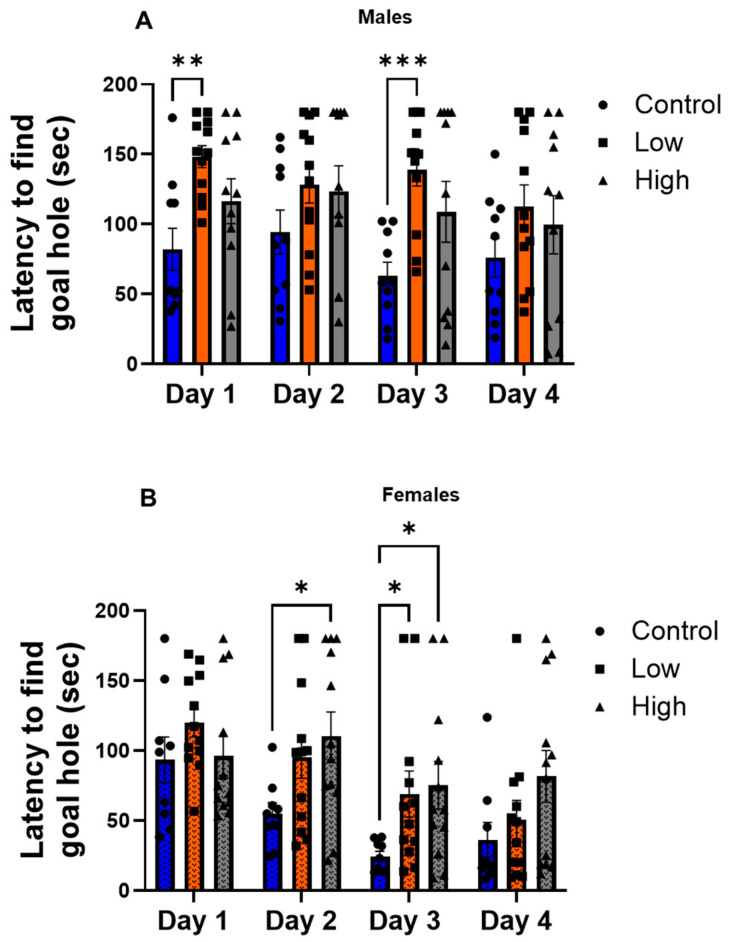
Spatial memory performance on the Barnes maze (mean ± SEM) for (**A**) males and (**B**) females. Animals were prenatally exposed to vehicle (Control) or IMI at either a low (0.5 mg/kg/day) or high (5.7 mg/kg/day) dose. Animals were given 4 trials/day for 4 days. Data represent the duration of seconds for the subject to locate the goal hole. * *p* < 0.05, ** *p* < 0.01, and *** *p* < 0.001. Symbols represent individual animals and circles, squares, and triangles represent control, low, and high dose animals, respectively. Sample sizes were 10–12 for males and 9–12 for females.

For males on the Barnes maze (Figure 1A; F [2.68, 80.5] = 3.56; *p* = 0.02; two-way repeated measures ANOVA with Dunnett’s multiple comparisons tests), low-dose IMI exposure significantly increased the latency to locate the goal hole on training days 1 (82.1 s vs. 148.3 s; *p* = 0.003) and 3 (63.1 s vs. 138.9 s; *p* = 0.0001) compared to controls. Latency was not affected for the high-dose group. For females (Figure 1B), high-dose exposure significantly increased latency on day 2 high (110.4 s) vs. controls (54.8; *p* = 0.02; Dunnett’s multiple comparisons test), and on day 3, latency to locate the goal hole was significantly longer for both high (75.3 s; *p* = 0.02) and low (69.1 s; *p* = 0.04) IMI-exposed subjects compared to controls (24.5 s). IMI exposure did not significantly affect the probe trial in males (H(2) = 2.028, *p* = 0.36) or females (H(2) = 1.12, *p* = 0.57, Kruskal–Wallis test) compared to controls (Supplementary Figure S1).

### 3.2. Both IMI-Exposed Males and Females Displayed Impairments in Procedural Learning

**Figure 2:**
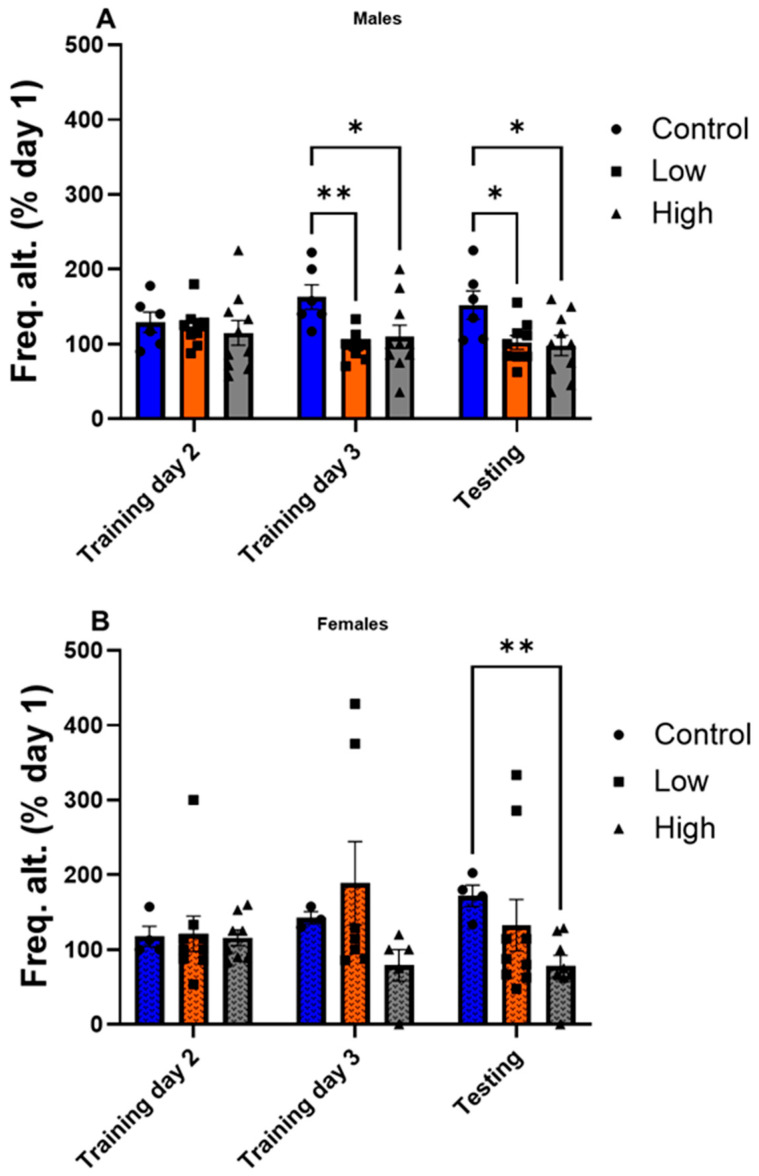
Procedural memory performance using forced alternation in the T-maze (mean ± SEM) for (**A**) males and (**B**) females. Animals were prenatally exposed to vehicle (Control) or IMI at either a low (0.5 mg/kg/day) or high (5.7 mg/kg/day) dose. Animals were given a forced choice, followed by 10 trials/day for 3 days and on testing day they were given 10 trials without a forced choice. Data are plotted as the frequency of correct alternations relative to Day 1. * *p* < 0.05, ** *p* < 0.01. Symbols represent individual animals and circles, squares, and triangles represent control, low, and high dose animals, respectively. Sample sizes were 9–10 for males and 4–9 for females.

IMI exposure reduced rewarded alternation in both males and females on the T-maze compared to controls. Interestingly, males appeared more sensitive to IMI than females as low and high IMI exposures had an impact compared to only high exposure in females. Specifically, in males (Figure 2A; F (2,22) = 3.50; Mixed-effects model with Tukey’s post-test), controls performed significantly better than both low- (*p* = 0.007) and high- (*p* = 0.029) dosed groups on day 3 of training and the testing day (con vs. low, *p* = 0.048, con vs. high, *p* = 0.026). In females (Figure 2B; F (4,30) = 2.73; mixed-effects model with Dunnett’s multiple comparisons test), high-dose individuals (78.5 ± 15.5% of baseline) performed worse than controls on the testing day (171.8 ± 16.6%; *p* = 0.003).

### 3.3. Prenatal IMI Increased Anxiety-like Behavior in Males but Not Females

**Figure 3:**
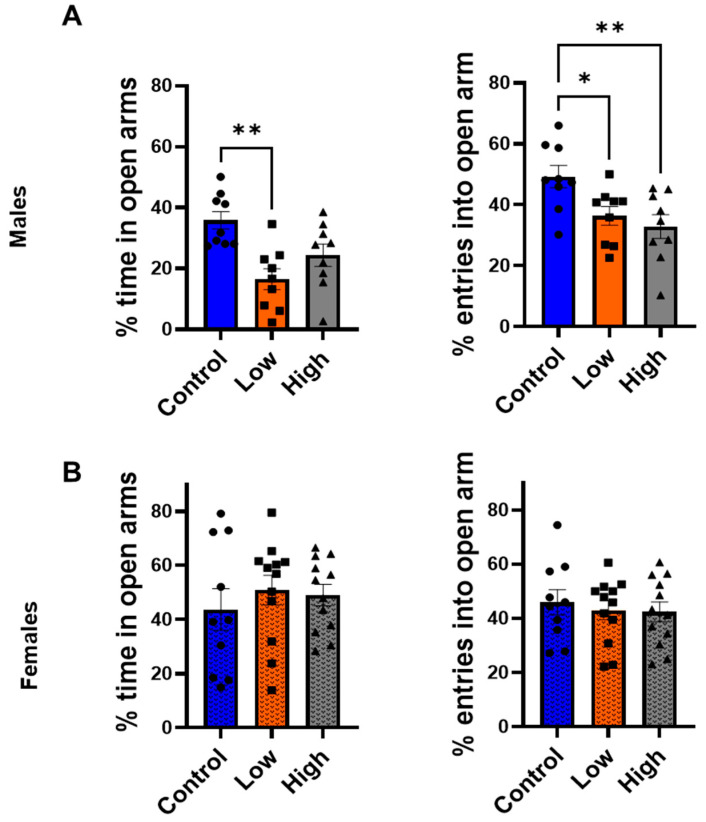
Anxiety-like behavior was tested using Elevated plus maze (EPM) (mean ± SEM) for (**A**) males and (**B**) females. We measured percent of time spent in the open arms and percent of entries into open arms. * *p* < 0.05, ** *p* < 0.01. Symbols represent individual animals and circles, squares, and triangles represent control, low, and high dose animals, respectively. Sample sizes were 9–10 for males and 10–12 for females.

Males displayed increased anxiety-like behavior in both the EPM and light/dark transition test, with greater effects observed in low-dose individuals compared to controls. In the EPM (Figure 3A), both high- (32.9 ± 3.9%, *p* = 0.009) and low-dose (36.4 ± 3.0%, *p* = 0.04) subjects made significantly fewer percentage of entries into open arms than controls (49.2 ± 3.7%; F (2,24) = 5.87; one-way ANOVA with Tukey’s post-tests), but only low-dose males (16.4 ± 3.4%) spent significantly less time than controls for the percent of total time spent in in the open arms (36.8 ± 2.8%, *p* = 0.001; F (2,24) = 8.48; one-way ANOVA with Tukey’s post-tests). No significant differences were detected in females in the EPM (Figure 3B) (F (2,31) = 0.2212, *p* = 0.8).

**Figure 4:**
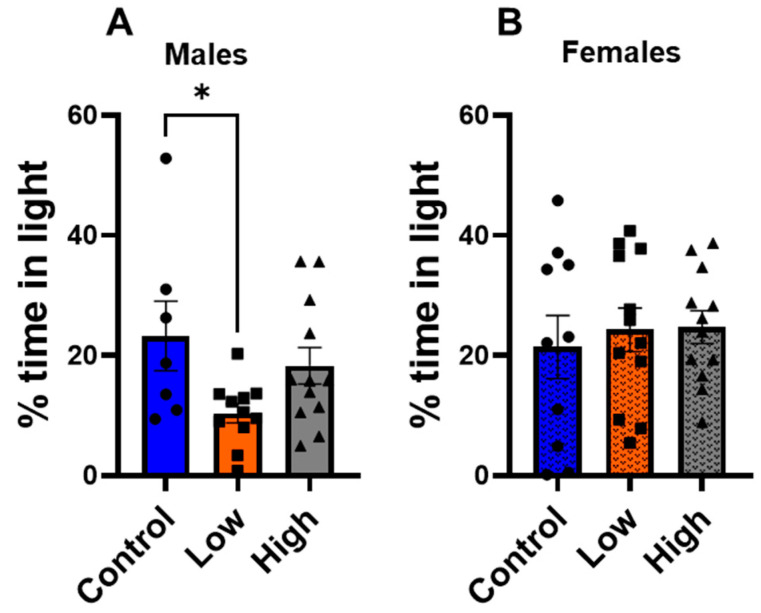
Anxiety-like behavior was tested in the light–dark transition test (mean ± SEM) for (**A**) males and (**B**) females. We measured percent of time spent in the light portion of the box. * *p* < 0.05. Symbols represent individual animals and circles, squares, and triangles represent control, low, and high dose animals, respectively. Sample sizes were 10–12 for males and 7–12 for females.

In the light/dark transition test (Figure 4), low-dose males (10.4 ± 1.6%) spent significantly less time in the illuminated half of the box than controls (25.3 ± 5.8%; F (2,27) = 3.59; *p* = 0.04; one-way ANOVA with Tukey’s post-test), but high-dose males were not statistically different from controls (18.3 ± 3.0%; *p* = 0.57). Again, IMI-exposed females did not differ from controls in this test (Figure 4B) (F (2,31) = 0.2068, *p* = 0.8).

### 3.4. Prenatal IMI Did Not Affect Depression-like Behavior in Either Sex

**Figure 5:**
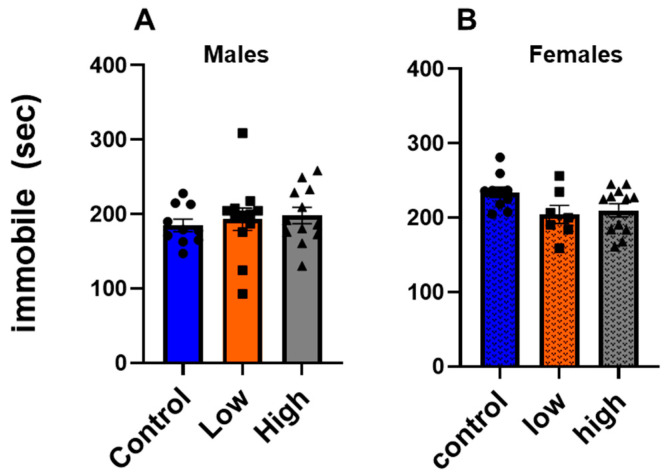
Depression-like behavior was tested in the tail suspension test for (**A**) males and (**B**) females. Data (average + SEM) is plotted as duration of time spent immobile (sec). Symbols represent individual animals and circles, squares, and triangles represent control, low, and high dose animals, respectively. Sample sizes were 10–12 for males and 7–12 for females.

In the tail suspension test (Figure 5), we did not find any effect of prenatal IMI exposure in either males (F (2,31) = 0.2915, *p =* 0.74) or females (F (2,26) = 2.824; *p* = 0.077, one-way ANOVA with Tukey’s post-tests).

### 3.5. Prenatal IMI Did Not Influence Working Memory in Either Sex

**Figure 6:**
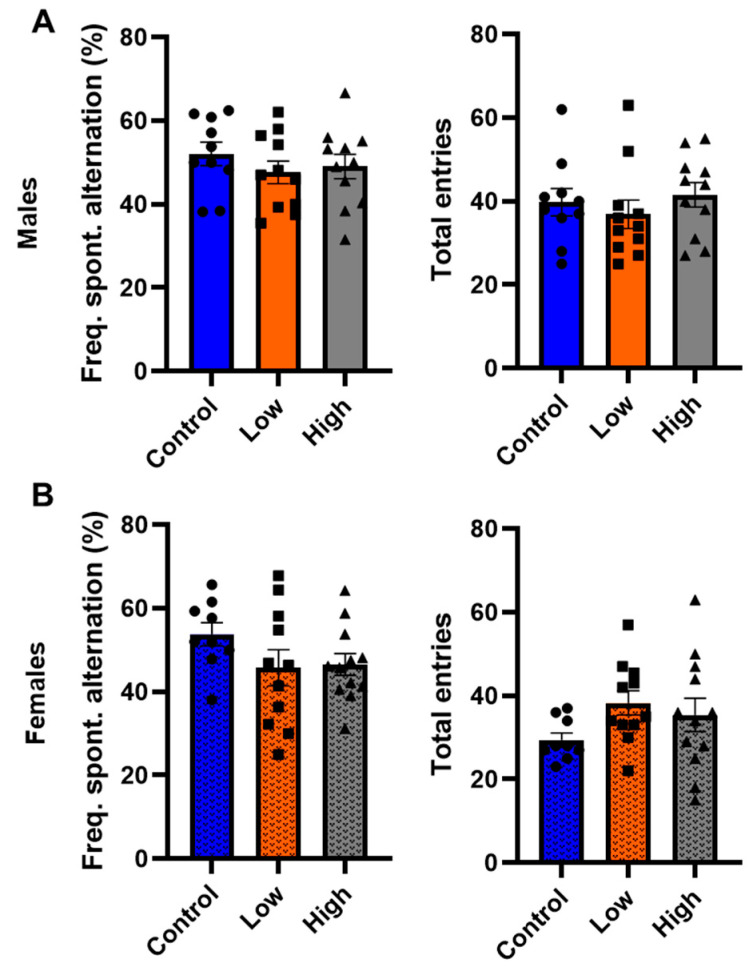
Working memory performance tested in the Y-maze (mean ± SEM) for (**A**) males and (**B**) females. Animals were prenatally exposed to vehicle (Control) or IMI at either a low (0.5 mg/kg/day) or high (5.7 mg/kg/day) dose. The percent of correct spontaneous alternation and the total number of entries were calculated for the last 8 min of a 10 min test. Symbols represent individual animals and circles, squares, and triangles represent control, low, and high dose animals, respectively. Sample sizes were 10–11 for males and 9–13 for females.

In the Y-maze, IMI exposure did not significantly alter the frequency of spontaneous alternations in either males (Figure 6A; F (2,28) = 0.60; *p* = 0.56; one-way ANOVA) or females (Figure 6B; F [(2,30) = 1.60; *p* = 0.22; one-way ANOVA). Additionally, IMI exposure did not significantly affect the number of entries for either males (Figure 6A; F (2,28) = 0.35; *p* = 0.71; one-way ANOVA) or females (Figure 6B; F (2,30) = 1.95; *p* = 0.22; one-way ANOVA) compared to controls.

### 3.6. Prenatal IMI Exposure Altered α7 and α4 nAChR Expression in Males but Not Females

**Figure 7:**
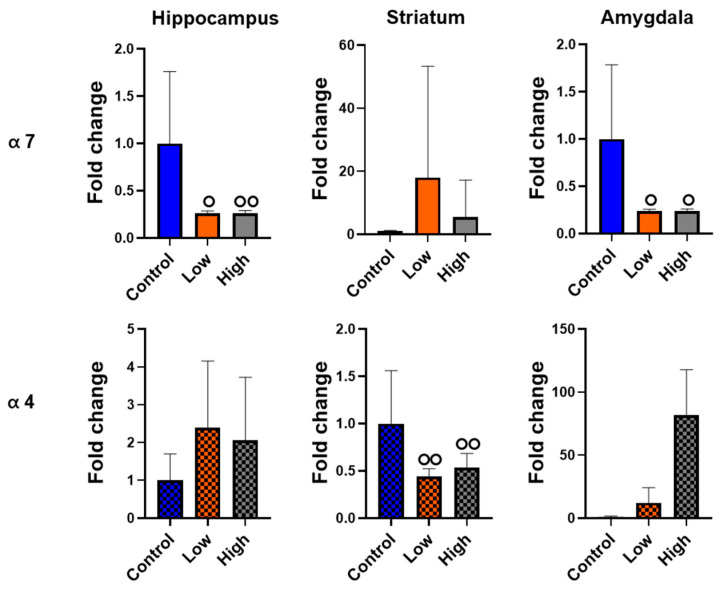
The relative expression of the α-7 subunit (**top**) and α-4 subunit (**bottom**) of nAChR in the hippocampus, striatum, and amygdala of male mice. Data are represented as mean +SEM and are expressed relative to controls. Data were analyzed with Cohen’s D; one circle indicates a medium effect and 2 circles indicate a relatively large effect size compared to controls. Sample sizes: n = 3 control, 5 low dose, 7 high dose.

**Figure 8:**
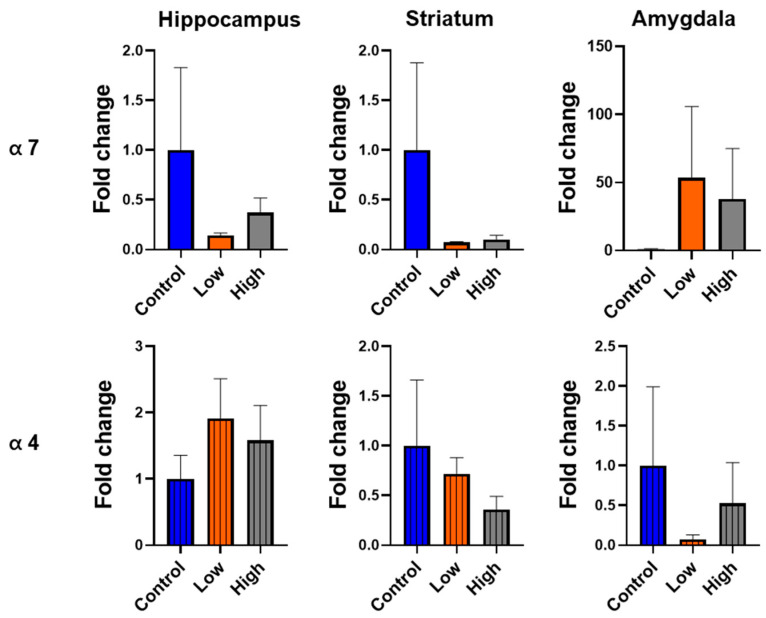
The relative expression of the α-7 subunit (**top**) and α-4 subunit (**bottom**) of nAChR in the hippocampus, striatum, and amygdala of female mice. Data are represented as mean +SEM and are expressed relative to controls. Data were analyzed with Cohen’s D. Sample sizes: n = 4 control, 6 low dose, 8 high dose.

We performed qPCR for α7 or α4 nAChR subunits in the hippocampus, striatum, and amygdala. For males, we did not find a statistical difference in any comparison using ANOVA. However, Cohen’s D effect size analysis revealed hippocampal α7 expression had both large (control vs. high dose; Cohen’s D = 0.86) and medium (control vs. low dose; Cohen’s D = 0.65) effect sizes, striatal α4 expression had large effect sizes for both prenatal IMI doses (Cohen’s Ds: control vs. low dose = 1.62; control vs. high dose = 1.13), and amygdala α7 expression had medium effect sizes for both doses (Cohen’s Ds: control vs. low dose = 0.59; control vs. high dose = 0.78; Figure 7). IMI exposure did not significant differences in the expression of either receptor in the brains of females when we analyzed the data with an ANOVA or with Cohen’s D effect size analysis (Figure 8).

## 4. Discussion

This study examined how prenatal exposure to relatively low doses of IMI affects anxiety- and depression-like behaviors, procedural memory, working memory, and spatial memory in male and female mice. We found spatial memory and procedural memory were impacted by prenatal IMI in both males and females (Figure 1 and Figure 2). Working memory, measured by the spontaneous alternation, was not altered by IMI (Figure 6) nor was depression-like behavior (Figure 5) compared to controls. However, anxiety-like behavior was impacted by prenatal IMI exposure, but only in males (Figure 3 and Figure 4). Interestingly, IMI affected nAChR expression in the brains of male but not female mice (Figure 7 and Figure 8), suggesting a potential mechanism underlying the observed behavioral differences, although this needs additional investigation. Previous studies have demonstrated that prenatal neonicotinoid exposure impairs sensorimotor processes [47,68,69], and our work demonstrates IMI effects on more demanding cognitive tasks. Additionally, we demonstrate that these effects of IMI can be produced solely from in utero exposure, demonstrating powerful lifelong consequences that occur from IMI exposure during a critical period of development.

Prenatal IMI exposure impairs multiple forms of learning as measured by decreased acquisition of spatial memory in the hippocampal-dependent Barnes maze. For both sexes, prenatal IMI exposure increased the time to locate the entry hole for 2 of the first 3 training days, but individuals had successfully learned the task by day 4. We did not see an effect of IMI exposure on all days of training, possibly due to individual variation in learning acquisition in a cognitively challenging task. No significant differences were observed in the probe trial, indicating that hippocampal-dependent short term reference memory was not significantly affected by IMI exposure [60]. Our data are consistent with reports that other neonicotinoids impact memory. Male, but not female mice exposed to clothianidin as adults spent an increased amount of time learning the location of an escape hole in the Barnes Maze task compared to controls indicating dysfunctions in memory [52]. Additionally, adult male rats exposed to the neonicotinoid acetamiprid (20 mg and 40 mg/kg/day) for 28 days spent more time finding the escape platform in the Morris Water maze when compared to females [70]. Our work uses a prenatal exposure paradigm and thus suggests neonicotinoid exposure at multiple ages can cause negative effects on memory.

Although IMI exposure affected hippocampal dependent spatial learning in the Barnes maze (Figure 1), it did not affect spontaneous alternation on the Y-maze, a hippocampal-dependent working memory task (Figure 6). This difference may reflect the lesser cognitive demand of the Y-maze task, as it does not require learning. This lack of an effect in the Y-maze is observed in other studies with neonicotinoids [33,71]. Although, our Y-maze results differ from those of Mudgal et al. [72] who found that exposure to IMI for 28 days in adult animals significantly reduced the frequency of spontaneous alternations in male Swiss albino mice. However, Mudgal et al. used a much higher concentration of IMI than the current study (45 mg/kg BW/day), and their control mice performed spontaneous alternations at a much higher rate (close to 80% vs. 52.1 ± 3.0% [males] and 53.8 ± 2.9% [females]), so it is unclear if the differences between our experiments reflect the difference between adult and prenatal exposure windows, dose, or differences in experimental details such as mouse strain.

In the striatum-dependent T-maze task, IMI exposure impaired performance for both sexes, but males appeared more strongly affected than females (Figure 2) as IMI-exposed males displayed impairments in both training and on the testing day, whereas females only showed impairments on the testing day. In contrast, in the Barnes maze, IMI-exposed subjects eventually reached the same level of competency as controls but needed more training than the controls to do so. Providing extra training on the T-maze would allow us to see if a similar pattern holds true for procedural learning and provide greater resolution into how IMI impairs learning.

Males were also more affected by prenatal IMI exposure in tests of anxiety-like behavior. In both the EPM (Figure 3) and light-dark transition task (Figure 4) males exposed to IMI had an increase in anxiety-like behavior compared to controls. This was not observed in females and is consistent with the findings of Kubo et al. [52]. A single dose of clothianidin (5 mg/kg and 50 mg/kg) increased anxiety-like behaviors as measured by reduced exploration in the Open Field test and reduced time and entries into open arms in the EPM in males but not in females [52]. We cannot determine from our data how this sex difference is produced, but it may involve differences in dopaminergic signaling in the amygdala. Male mice have significantly more dopaminergic boutons in the basolateral amygdala than female mice [73], and nAChR activation enhances dopamine release in the amygdala [74], so males may experience greater IMI-induced dysregulation of this signaling than females. The cholinergic system also plays a role in modulating anxiety and there are sex differences with respect to nAChR expression and acetylcholine release, thus this system is another potential target for IMI [75,76,77]. Alternatively, recent studies suggest that male rats generally demonstrate greater anxiety-like behavior than females on the EPM [78], so it may be that our experiments using females did not adequately capture intergroup differences in anxiety. Finally, another potential mechanism could be sex differences in detoxifying enzymes. The P450 enzymes play a role in neonicotinoid resistance in insects [79,80] and there are known sex differences in the expression of specific enzymes in house flies [81] and rodents [82]. For example, the brains of male rats express higher mRNA for *Cype2c11*, *Cyp2c13*, *Cyp1a1*, and *Cyp4a3* compared to females, and this may be a mechanism underlying the outcomes in the current study [82].

Our EPM and light–dark transition data also suggest IMI follows a non-linear dose response curve with respect to male anxiety-like behaviors. Only the low-dose IMI group displayed differences on the light-dark transition task (Figure 4), and the low-dose IMI group displayed differences on both parameters in the EPM (Figure 3), whereas the high-dose IMI group only displayed differences for percent of entries into open arms. It is well documented that environmental chemical exposure can produce U-shaped dose–response curves with low doses having different effects than relatively high doses [83,84]. We hypothesize that this non-linear dose effect occurs with respect to IMI, but this requires additional testing. Further, chemicals including IMI exert different effects in different tissues, cell types, and brain regions [26,58,85].

We found reduced mRNA transcript expression of the nAChR α7 in the hippocampus and amygdala of males, and a reduction in α4 transcript in the striatum of males. The reduced expression of these receptors in the male tissues mirrors the effects produced by prenatal nicotine exposure [45,86], and may reflect homeostatic responses to excessive activation of nAChRs in the prenatal period. This reduced expression may be partially responsible for the learning deficits in males. However, our data are correlational, and further mechanistic studies are needed. The relationship between α7 expression in the amygdala and increased anxiety-like behavior is less clear, however, as reductions in cholinergic signaling typically reduce these behaviors [87,88]. However, these data do not necessarily indicate a lack of nAChR-related effects on cognition. Receptor expression is only one component of neurotransmission. Endogenous acetylcholine release, currents evoked by acetylcholine binding, cholinesterase activity, intrinsic excitability of nAChR-expressing neurons, and postsynaptic neurotransmitter release can all be altered by IMI exposure [71,89]. Changes to any of these could cause increased anxiety-like behavior in males and could also influence procedural and spatial learning.

The lack of effects of IMI on female neural α7 and α4 subunit expression are puzzling given that females demonstrated some impairments in the learning tasks. One explanation is that IMI alters other nAChRs; whereas α7 and α4β2 are heavily implicated in learning and memory, other isoforms of the receptors exist [90]. Additionally, IMI may affect the plasticity of nAChR expression (i.e., the ability to change nAChR expression as an individual learns a task). IMI may affect cognition by mechanisms such as altering the endocrine system indirectly leading to changes in cognition. Additionally, numerous other pesticides have multiple pharmacological effects beyond their intended effects. For example, organophosphates, designed to prevent cholinergic degradation at the synapse, also enhance protein kinase A activity [91,92]. Modeling studies show a single toxicant can have strong affinity for dozens of proteins in a single organ [93], so it would not be surprising if a similar phenomenon occurs with IMI exposure in the brain. However, it is important to note the small sample size in the qPCR studies, and we did not quantify the receptor proteins. Other limitations in our work include the potential for dams to alter their maternal care or have heightened stress in response to treatment with IMI.

Future work could investigate the impact of IMI exposure on other candidates such as detoxifying P450 enzymes [82] and markers of neuronal proliferation [71], or mechanistic studies of receptor pharmacology and assays of neurotransmitters. Ultimately, it is likely that the effects of IMI derive from some combination of the above hypotheses and dysregulation of nAChR-mediated signaling [71]. Determining these mechanisms will be essential, as this insight will be necessary to develop therapies to combat IMI’s behavioral effects.

## 5. Conclusions

This study provides additional evidence that prenatal exposure to IMI at relatively low doses can impact anxiety-like behavior and cognition and that sex differences exist in response to prenatal IMI exposures. The effects of neonicotinoids on nAChR-mediated signaling require additional exploration to identify mechanistic insight into these effects within the brain.

## Data Availability

All relevant data of this study are given in the manuscript. Additional data will be provided upon request.

## Supplementary Materials

The following supporting information can be downloaded at: https://www.mdpi.com/article/10.3390/toxics13110918/s1, Figure S1: Spatial memory performance on the probe trial for the Barnes maze (mean +SEM) for males and females. Animals were prenatally exposed to vehicle (Control) or IMI at either a low (0.5 mg/kg/day) or high (5.7 mg/kg/day) dose. Animals were tested on the Barnes maze for 4 days followed by the probe trial on the 5th day. Data represent the duration of seconds for the subject to locate the goal hole. Symbols represent individual animals and circles, squares and triangles represent control, low, and high dose animals, respectively. Sample sizes were 10–12 for males and 9–12 for females.

## Abbreviations

The following abbreviations are used in this manuscript:

EPM	Elevated Plus Maze
IMI	Imidacloprid
nAChR	Nicotinic Acetylcholine Receptors
NOAEL	No Observable Adverse Effects Level
SCM	Sweetened Condensed Milk
