# Supplementary material for: Prenatal Exposure to Imidacloprid Affects Cognition and Anxiety-Related Behaviors in Male and Female CD-1 Mice
Source: Toxics. 2025 Oct 27;13(11):918. doi: 10.3390/toxics13110918 (PMC12656406; doi:10.3390/toxics13110918)
Supplement: Supplementary file 1 [file toxics-13-00918-s001.zip › toxics-3942951-supplementary.pdf]

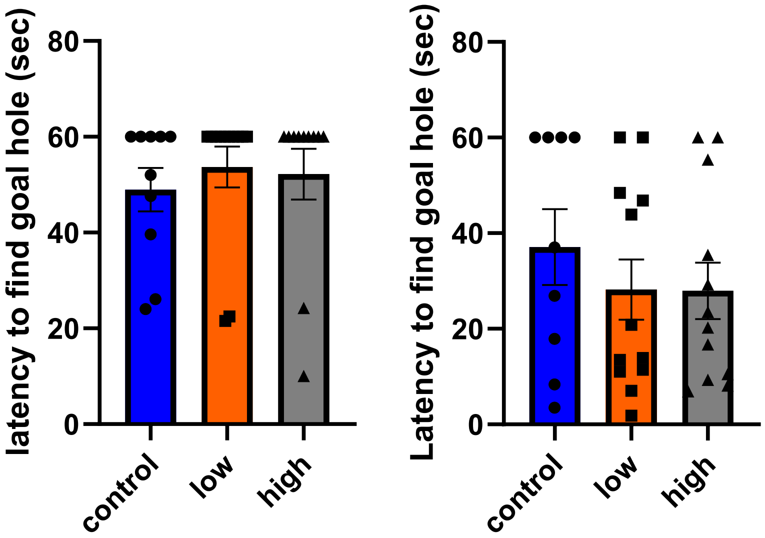

Supplementary Figure S1. Spatial memory performance on the probe trial for the Barnes maze (mean ±SEM) for males and females. Animals were prenatally exposed to vehicle (Control) or IMI at either a low (0.5 mg/kg/day) or high (5.7 mg/kg/day) dose. Animals were tested on the Barnes maze for 4 days followed by the probe trial on the 5th day. Data represent the duration of seconds for the subject to locate the goal hole. Symbols represent individual animals and circles, squares and triangles represent control, low, and high dose animals, respectively. Sample sizes were 10-12 for males and 9-12 for females
